# Critical analysis of the “nanoleakage concept” in endodontics: a narrative review

**DOI:** 10.3389/fdmed.2026.1880780

**Published:** 2026-09-21

**Authors:** José Luis Álvarez-Vásquez, Daniel Chavarría-Bolaños

**Affiliations:** 1 Discipline of Endodontics, Faculty of Dentistry, University of Cuenca, Cuenca, Ecuador; 2 Endodontics Postgraduate Program, Universidad de Costa Rica, San José, Costa Rica; 3 National Laboratory of Nanotechnology (LANOTEC), Centro Nacional de Alta Tecnología, San José, Costa Rica

**Keywords:** endodontics, microleakage, nanoleakage, retrograde filling, root canal filling, sealing ability, biomaterials, dentin

## Abstract

**Background:**

Microleakage models have historically defined leakage assessment in endodontics despite well-recognized methodological limitations, leading to increasing restrictions on their publication. Since the seminal description of the term nanoleakage, interest in nanoscale interfacial leakage has progressively expanded into endodontics, where achieving a bacteria-free seal is critical for treatment success.

**Objective:**

We aim to map and critically analyze the current evidence on nanoleakage in endodontics, identifying research trends, methodological approaches, and existing knowledge gaps across different clinical applications.

**Methods:**

A narrative review was conducted following a literature search to identify studies addressing nanoleakage within endodontic contexts. Eligible studies were screened and categorized according to their clinical application and methodological approach.

**Results:**

Evidence is derived from *in vitro* studies, mostly using a qualitative approach, with significant heterogeneity in experimental designs and interpretation criteria. Most studies rely on passive tracer-based methodologies, which may not adequately reflect the dynamic behavior of fluids and bacterial by-products at the nano-interface. Research was primarily focused on areas including the bonding of adhesives to coronal and radicular dentin as well as in retrograde filling and root canal filling.

**Conclusions:**

Understanding nanoleakage is an important objective, yet endodontic models remain underdeveloped. The existing evidence is limited by methodological inconsistencies and a lack of clinical validation. Future research should prioritize the development of dynamic, clinically relevant models to better understand interfacial sealing and improve the predictability of endodontic outcomes.

## 1 Introduction

In the endodontic context, coronal and apical seals are critical for preventing microbial and microbial by-product leakage (1). Indeed, pulpal irritation is more likely caused by microbial insult than by the chemical toxicity of restorative materials (2), and bacteria are the primary etiology of apical periodontitis (3, 4). Historically, microleakage (ML) studies have been the most reported and published model for evaluating leakage in endodontics and operative dentistry, employing numerous substances and techniques (5–9). In 1976, ML was defined as “the passage of bacteria, fluids, molecules or ions between a cavity wall and the restorative material applied to it” (5), giving rise to increased research, particularly in operative dentistry and endodontics. However, ML studies have inherent drawbacks and disadvantages, and their publication has even been restricted in several endodontic journals (10, 11).

Adhesive dentistry has dramatically changed since Buonocore introduced the enamel acid-etch technique in 1955 (12), with thousands of restorative and non-restorative esthetic treatments performed daily, globally, thanks to dental adhesives (13). The endodontic field has also taken advantage of adhesive science, although some limitations in bonding performance with coronal dentin (14) and radicular dentin (15) have been described. Many studies have confirmed that no existing adhesive systems are capable of preventing ML in the long term (16–18). However, as mentioned above, ML studies are controversial. A recent manuscript (19) drew attention to the fact that thirty years have passed since Sano et al. coined the term nanoleakage (NL) in adhesive dentistry in their seminal articles in 1995 (20, 21). NL is leakage that, even in the presence of gap-free restorations and sound clinical practice, occurs through submicron spaces (20-100 nm) within incompletely infiltrated hybrid layers, thus weakening the adhesive interface (20, 21). NL studies have garnered interest in the endodontic field in the last few years.

However, to our knowledge, there is no study in the literature that analyzes, discusses, and integrates this research. With this narrative review, we aim to provide a comprehensive analysis of the current evidence and challenges faced using NL models in different endodontic topic areas, like the bonding of adhesives to coronal and radicular dentin, orifice barriers, as well as in retrograde and root canal filling.

## 2 Methods

### 2.1 Study design

The aim of this critical narrative review was to analyze the existing literature on NL within the field of endodontics, identifying not only research trends but also knowledge gaps in this area.

### 2.2 Search strategy

Articles were screened using the PubMed database, from database inception to 30 July 2026, using the following search strategy: "nanoleakage"[All Fields] AND ("endodontal"[All Fields] OR "endodontic"[All Fields] OR "endodontical"[All Fields] OR "endodontically"[All Fields] OR "endodontics"[MeSH Terms] OR"endodontics"[All Fields]). All studies retrieved using the search terms “nanoleakage AND endodontics” were considered for inclusion. Then the search was done using the SCOPUS database, and finally, by using an automatic bibliographic search (22), Google Scholar (via SerpAPI), EuropePMC, Semantic Scholar and CrossRef.

### 2.3 Eligibility criteria and study selection

All articles containing the terms “nanoleakage” and “endodontics” in their titles, abstracts, or keywords were considered for analysis. Publications were required to be written in English, and the full text had to be available. Case reports and secondary research studies (such as meta-analyses or systematic reviews) were excluded, as were those conducted on primary teeth. The titles, abstracts, and keywords were initially screened, followed by a full-text assessment of the preselected articles. Relevant data were then extracted and summarized in a Microsoft Excel spreadsheet for data processing. Screening and selection were performed independently by two reviewers, and any disagreements were resolved through discussion.

### 2.4 Data analysis

A descriptive analysis of the included studies was performed. The following variables were evaluated: publication year, journal, country of origin according to the corresponding author's affiliation, study design, type of NL experiment reported, and the variables analyzed. Additionally, studies were categorized according to their focus on NL in: (i) coronal adhesive restorations under simulated pulpal pressure (SPP), (ii) retrograde fillings, (iii) root canal fillings, (iv) bonding of adhesives or luting cements to radicular dentin and (v) orifice barriers.

## 3 Results

A total of 216 potential articles were identified during the initial screening. After the exclusion of duplicates, articles which full text was not available, and after excluding meta-analysis, systematic reviews, scoping reviews, or if the original research was not addressed to endodontics context or if was focused on primary teeth, a final number of 45 articles were identified (Figure 1). The publication period ranged from 2005 to 2026. Geographically, most of the articles were originated from Brazil (37.8%), the United States (13.3%) and Italy (11.1%) (Figure 2).

**Figure 1:**
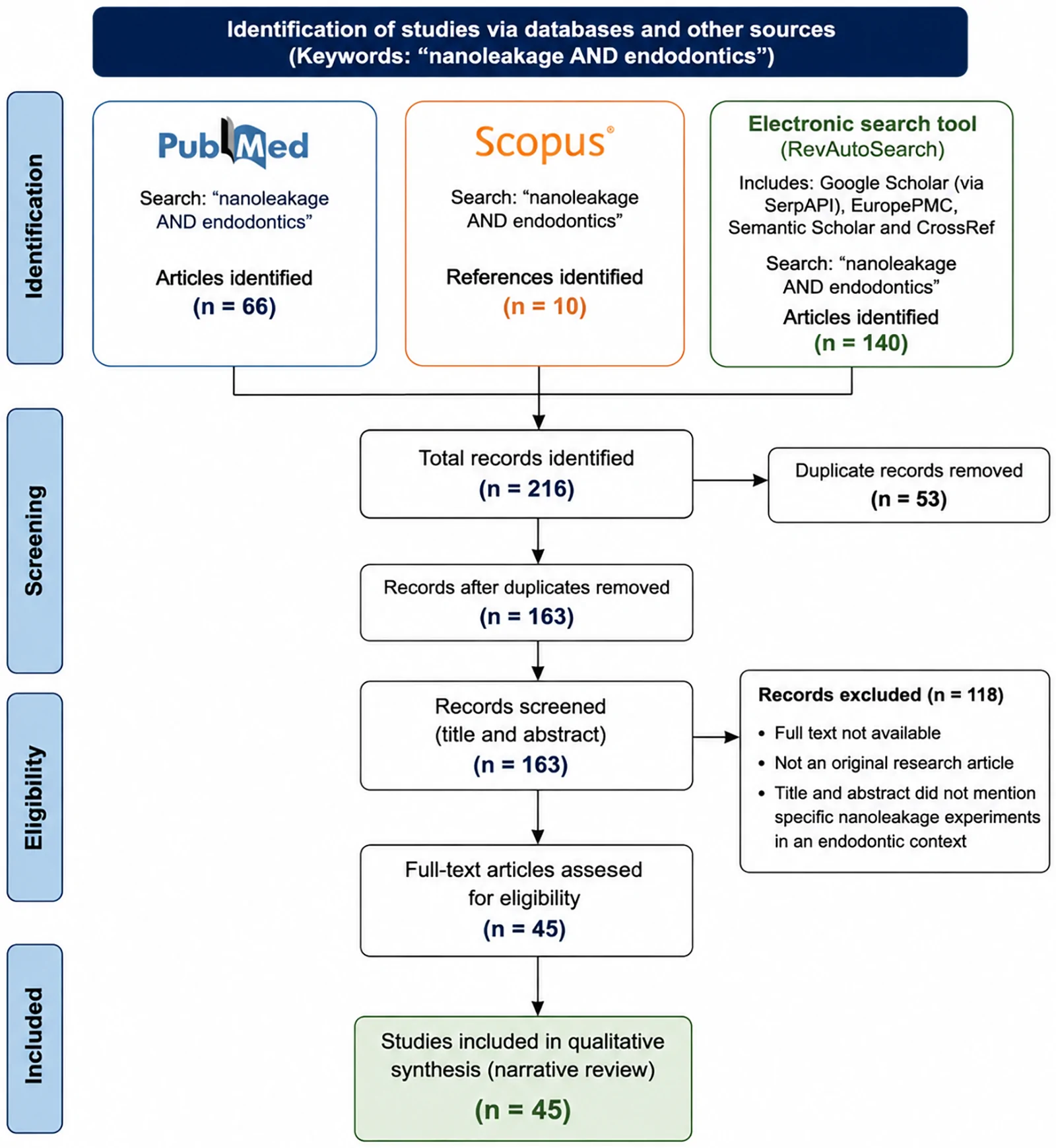
Prisma flowchart of the article's selection process.

**Figure 2:**
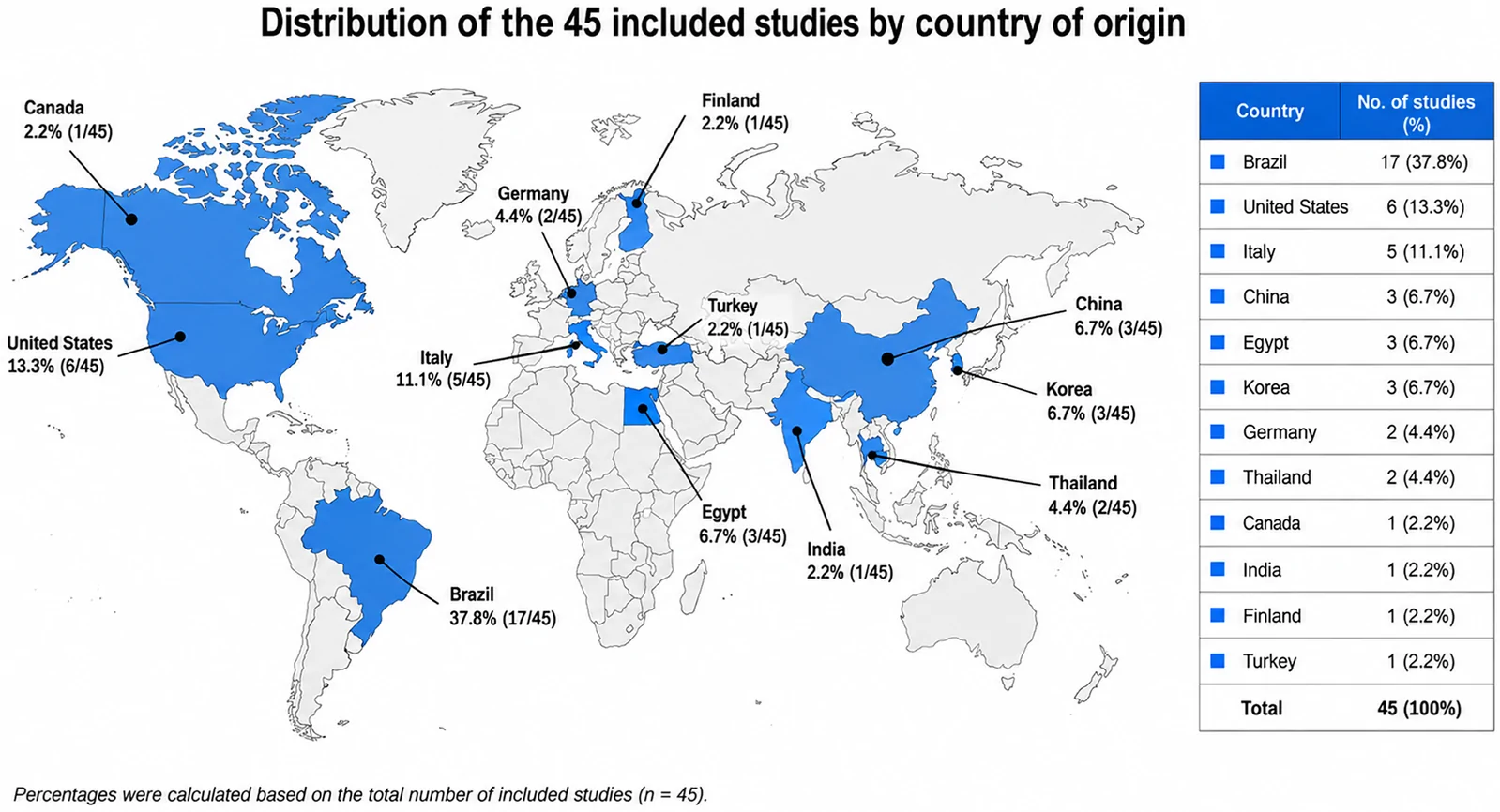
Distribution of publications by country.

The three journals with the highest number of publications were the Journal of Adhesive Dentistry (*n* = 9), the Journal of Endodontics (*n* = 6) and Operative Dentistry (*n* = 5). All 45 included studies were conducted *in vitro* using extracted teeth as experimental substrates. Regarding the methodological approach, 41 studies (91.11%) relied exclusively on qualitative assessment of NL, three (6.67%) used quantitative pressurized liquid-flow models, and one (2.22%) employed a combined qualitative and quantitative approach. Among the 41 studies using exclusively qualitative methods, silver nitrate was the predominant tracer (*n* = 40; 97.56%), whereas only one study (2.44%) used a lanthanum nitrate. Tracer penetration was most frequently assessed by scanning electron microscopy (SEM), (*n* = 29; 70.73%), followed by transmission electron microscopy (TEM) (*n* = 5; 12.20%), light microscopy (*n* = 3; 7.32%), and micro-CT (*n* = 2; 4.88%). Two additional studies (4.88%) combined SEM and TEM analyses. The three studies employing quantitative pressurized liquid-flow models did not rely on microscopic tracer assessment. In addition, one study used a mixed methodological approach, combining qualitative environmental scanning electron microscopy (ESEM) in backscattered-electron mode with quantitative energy-dispersive x-ray analysis (EDAX) to determine the amount of silver within the specimens.

Regarding the endodontic application investigated, most studies evaluated NL associated with bonding agents or luting cements in radicular dentin (*n* = 27; 60.00%). Nine studies (20.00%) investigated root canal filling materials, five (11.11%) evaluated restorative material–adhesive interfaces in coronal dentin under SPP, two (4.44%) investigated retrograde filling materials, and two (4.44%) evaluated orifice barriers. The characteristics and methodological distribution of the 45 included studies (23–67) are summarized in Table 1.

**Table 1:** Article analysis by title, year of publication, country of origin, corresponding author, journal, study design and nanoleakage analysis method.

Title	Year	Country	Corresponding author	Journal	Study design	Nanoleakage analysis method	Bonding of adhesives to coronal dentin—with simulated pulpal pressure (SPP)	Retrograde filling	Root canal filling	Bonding of adhesives or luting cements to radicular dentin	Orifice Barriers
Nano-leakage and Fracture Resistance of Bioceramic Coronal Plug Materials in Immature Teeth: A Micro-CT and Finite Element Analysis (23)	2026	India	Kavalipurapu Venkata Teja	Giornale Italiano di Endodonzia	*In vitro*	Micro-CT/silver nitrate					**X**
Influence of Natural Dentin Biomodification Agent on Push-Out Bond Strength and Nanoleakage of Self- Adhesive Resin Cement Luting of Glass- Fiber Posts (24)	2025	United States	Tainah Oliveira Rifane	Journal of Esthetic and Restorative Dentistry	*In vitro*	SEM/silver nitrate				**X**	
A comparison of different cleaning approaches for blood contamination after curing universal adhesives on the dentine surface (25)	2024	China	Chen Chen	Dental Materials	*In vitro*	SEM/silver nitrate				**X**	
Nanoleakage of apical sealing using a calcium silicate-based sealer according to canal drying methods (26)	2024	Korea	Jin-Woo Kim	Restorative Dentistry and Endodontics	*In vitro*	Pressure liquid flow device			**X**		
A Study on Nanoleakage of Apical Retrograde Filling of Premixed Calcium Silicate-Based Cement Using a Lid Technique (27)	2024	Korea	Yoon Lee	Materials	*In vitro*	Pressure liquid flow device		**X**			
Long-term Evaluation of Dentin Bonding Properties of the Photoinitiator System Contained in Universal Adhesives Used in Fiber-Post Luting Procedures (28)	2023	Brazil	Alessandro Dourado Loguercio	Journal of Adhesive Dentistry	*In vitro*	SEM/silver nitrate				**X**	
Evaluation of Er YAG and Er,Cr YSGG lasers on intraradicular bonding using modified glass fiber tips (29)	2023	Finland	Jaana Hannele Sippus	Dental Materials Journal	*In vitro*	SEM/silver nitrate				**X**	
Effect of irrigation protocols on smear layer removal, bond strength and nanoleakage of ﬁber posts using a self-adhesive resin cement (30)	2023	Brazil	Giovana Mongruel Gomes	Restorative Dentistry and Endodontics	*In vitro*	SEM/silver nitrate				**X**	
Effect of dentin moisture on the adhesive properties of luting fiber posts using adhesive strategies (31)	2023	Brazil	Giovana Mongruel Gomes	Brazilian Oral Research	*In vitro*	SEM/silver nitrate				**X**	
Apical Sealing and Bioactivity of an Experimental Gutta-Percha Containing Niobium Phosphate Bioglass (32)	2023	Brazil	Edilausson Moreno Carvalho	Polymers	*In vitro*	SEM and TEM/lanthanum nitrate			**X**		
Delayed light activation of resin composite affects the bond strength of adhesives under dynamic simulated pulpal pressure (33)	2022	Thailand	Pipop Saikaew	Clinical Oral Investigations	*In vitro*	SEM/silver nitrate	**X(SPP)**				
Effects of dentin surface preparations on bonding of self-etching adhesives under simulated pulpal pressure (34)	2021	Thailand	Pipop Saikaew	Restorative Dentistry and Endodontics	*In vitro*	SEM/silver nitrate	**X(SPP)**				
Effects of various thicknesses and levels of mineral trioxide aggregate coronal plugs on nanoleakage and fracture resistance in revascularization: An *in vitro* study (35)	2021	Turkey	Tugba Bezgin	Australian Endodontic Journal	*In vitro*	micro-CT/silver nitrate					**X**
Influence of proanthocyanidins combined with ethanol-wet bonding on the bonding to root dentine quality of fibre posts to root dentine (36)	2020	China	Qingsong Jiang	European Journal of Oral Sciences	*In vitro*	FE-SEM/ silver nitrate				**X**	
Influence of alternative restoration technique with different composite resins for flared root reinforcement (37)	2020	Brazil	Giovana Mongruel Gomes	Journal of Adhesive Dentistry	*In vitro*	SEM/silver nitrate				**X**	
Effect of Crosslinked Chitosan Nanoparticles on the Bonding Quality of Fiber Posts in Root Canals (38)	2020	Canada	Anil Kishen	Journal of Adhesive Dentistry	*In vitro*	SEM/silver nitrate				**X**	
Effect of the Photo-initiator System Contained in Universal Adhesives on Radicular Dentin Bonding (39)	2020	Brazil	Alessandro Dourado Loguercio	Operative Dentistry	*In vitro*	SEM/silver nitrate				**X**	
Real-time nanoleakage and the flow characteristics of calcium silicate root canal filling materials (40)	2020	Korea	WooCheol Lee	Journal of the Mechanical Behavior of Biomedical Materials	*In vitro*	Pressure liquid flow device			**X**		
Bonding properties of universal adhesives to root canals prepared with different rotary instruments (41)	2019	Brazil	Giovana Mongruel Gomes	Journal of Prosthetic Dentistry	*In vitro*	SEM/silver nitrate				**X**	
Effect of different adhesive strategies on bond quality of fiber posts cemented in endodontically treated teeth (42)	2019	Brazil	Giovana Mongruel Gomes	Iranian Endodontic Journal	*In vitro*	SEM/silver nitrate				**X**	
Adhesive Durability Inside the Root Canal Using Self-adhesive Resin Cements for Luting Fiber Posts (43)	2017	Germany	Kerstin Bitter	Operative Dentistry	*In vitro*	SEM/silver nitrate				**X**	
Effect of Dentin Moisture and Application Mode of Universal Adhesives on the Adhesion of Glass Fiber Posts to Root Canal (44)	2017	Brazil	Giovana Mongruel Gomes	Journal of Adhesive Dentistry	*In vitro*	SEM/silver nitrate				**X**	
Effect of sonic application of Universal Adhesive systems on bond strength of fiber posts to root canal (45)	2016	Brazil	Cesar Augusto Galvao Arrais	Journal of Adhesive Dentistry	*In vitro*	SEM/silver nitrate				**X**	
Effect of dentin moisture on cementation of fiber posts to root canals (46)	2016	Brazil	Alessandro Dourado Loguercio	Journal of Adhesive Dentistry	*In vitro*	SEM/silver nitrate				**X**	
Effects of immediate dentin sealing and pulpal pressure on resin cement bond strength and nanoleakage (47)	2016	Brazil	Andre F. Reis	Operative Dentistry	*In vitro*	SEM/silver nitrate	**X (SPP)**				
Influence of Delivered Radiant Exposure Values on Bonding of Fiber Posts to Root Canals (48)	2015	Brazil	Alessandro Dourado Loguercio	Journal of Adhesive Dentistry	*In vitro*	SEM/silver nitrate				**X**	
Bond degradation resistance of self adhesive sealer bonded to radicular dentin using an alternative adhesive strategy (49)	2015	Egypt	M.I. Riad	Tanta Dental Journal	*In vitro*	ESEM/Silver nitrate			**X**		
Effects of Sonic Application of Adhesive Systems on Bonding Fiber Posts to Root Canals (50)	2014	Brazil	Alessandro Dourado Loguercio	Journal of Endodontics	*In vitro*	SEM/silver nitrate				**X**	
Effect of chlorhexidine and ethanol-wet bonding with a hydrophobic adhesive to intraradicular dentine (51)	2014	China	Cynthia Kar Yung Yiu	Journal of Dentistry	*In vitro*	FE-SEM/silver nitrate				**X**	
Fracture Strength and Nanoleakage of Weakened Roots Reconstructed Using Relined Glass Fiber–Reinforced Dowels Combined with a Novel Prefabricated Core System (52)	2014	Egypt	Mona H. Mandour	Journal of Prosthodontics	*In vitro*	qualitatively: SEM/silver nitrate. Quantitative: EDAX (amount of silver nitrate identification)				**X**	
Effect of long-term simulated pulpal pressure on the bond strength and nanoleakage of resin-luting agents with Different Bonding Strategies (53)	2014	Brazil	Andre F Reis	Operative Dentistry	*In vitro*	SEM and TEM/silver nitrate	**X (SPP)**				
Two methods to simulate intrapulpal pressure effects upon bonding performance of self-etch adhesives (54)	2014	Brazil	V. P. Feitosa	International Endodontic Journal	*In vitro*	SEM/silver nitrate	**X (SPP)**				
Interfacial nanoleakage and bonding of self-adhesive systems cured with a modified-layering technique to dentin (55)	2013	Egypt	Enas Mobarak	Operative Dentistry	*In vitro*	FE-SEM/silver nitrate				**X**	
Aging Affects the adhesive interface of post luted with self-adhesive cements: a 1 year study (56)	2013	Italy	Lorenzo Breschi	Journal of Adhesive Dentistry	*In vitro*	Light Microscope/ silver nitrate				**X**	
Back-Scattered Electron Imaging for Leakage Analysis of Four Retrofilling Materials (57)	2012	Brazil	Ricardo Abreu Da Rosa	Microscopy Research and Technique	*In vitro*	SEM/silver nitrate		**X**			
Retentive strength and sealing ability of new self-adhesive resin cements in fiber post luting (58)	2011	Italy	Cecilia Goracci	Dental Materials	*In vitro*	SEM/silver nitrate				**X**	
Nanoleakage of Luting Agents for Bonding Fiber Posts After Thermomechanical Fatigue (59)	2011	Germany	Kerstin Bitter	Journal of Adhesive Dentistry	*In vitro*	FE-SEM/silver nitrate				**X**	
Influence of Luting Material Filler Content on Post Cementation (60)	2009	Italy	Lorenzo Breschi	Journal of Dental Research	*In vitro*	Light Microscope/ silver nitrate				**X**	
Bonding of Self-adhesive (Self-etching) Root Canal Sealers to Radicular Dentin (61)	2009	United States	Frankling R. Tay	Journal of Endodontics	*In vitro*	TEM/silver nitrate			**X**		
The Efficacy of Different Sealer Removal Protocols on Bonding of Self-etching Adhesives to AH Plus–contaminated Dentin (62)	2009	United States	Frankling R. Tay	Journal of Endodontics	*In vitro*	TEM/silver nitrate			**X**		
Push-out stress for fibre posts luted using different adhesive strategies (63)	2009	Italy	Lorenzo Breschi	European Journal of Oral Sciences	*In vitro*	Light Microscope/ silver nitrate				**X**	
Ultrastructure of Intraradicular Dentin After Irrigation with BioPure MTAD. II. The Consequence of Obturation with an Epoxy Resin-Based Sealer (64)	2006	United States	David H. Pashley	Journal of Endodontics	*In vitro*	TEM/silver nitrate			**X**		
Improving the Quality of the Quartz Fiber Postcore Bond Using Sodium Ethoxide Etching and Combined Silane/Adhesive Coupling (65)	2006	Italy	Francesca Monticelli	Journal of Endodontics	*In vitro*	SEM/silver nitrate				**X**	
Application of biologically-oriented dentin bonding principles to the use of endodontic irrigants (66)	2005	United States	F.R. Tay	American Journal of Dentistry	*In vitro*	TEM/silver nitrate			**X**		
Effectiveness of resin-coated gutta-percha cones and a dual-cured, hydrophilic methacrylate resin-Based Sealer in Obturating Root Canals (67)	2005	United States	David H. Pashley	Journal of Endodontics	*In vitro*	TEM/silver nitrate			**X**		

## 4 Discussion

This narrative review highlights the importance of recognizing that achieving complete control of NL in endodontics remains an ideal yet currently unattained goal. Although the search strategy was intentionally limited to the terms NL and endodontics, this decision was made to characterize the body of literature that is immediately retrievable by researchers performing an initial search on this topic. Interestingly, this approach demonstrated two complementary phenomena: some potentially relevant studies were not identified because they did not explicitly use the term NL whereas other retrieved publications did not actually evaluate NL despite being captured by the search strategy. These findings emphasize the current lack of conceptual and terminological standardization in this research area and should be considered when interpreting the available literature. As discussed earlier, the concept of NL arises from the understanding that the nanoenvironment comprises particles smaller than those typically evaluated; therefore, the potential for clinically relevant leakage cannot be disregarded. In this context, the traditional reliance on dyes, bacteria, or organic fluids as leakage markers is challenged by the recognition that the filtration of ions, endotoxins, viruses, and even gases may play an equally significant role. Although some clinical considerations of ML have been recognized (68) and valued (69), acknowledging the existence of NL suggests a possible conceptual change in endodontics, emphasizing the need to critically reassess the performance of current techniques and materials. An important distinction should be considered when interpreting the current evidence. Although the limitations identified in existing “nanoleakage” studies highlight the need for improved experimental methodologies, they should not be interpreted as indirect evidence supporting the clinical significance of the NL concept itself. Rather, they demonstrate that current laboratory models remain insufficient to establish whether NL as observed microscopically, is associated with biologically meaningful or clinically relevant outcomes.

Consequently, the principal challenge for future research should not simply be to demonstrate the presence or absence of NL, but to determine its clinical relevance through experimental models that move beyond passive tracer penetration; considering standardized aging protocols, functional permeability assessments and dynamic fluid transport. Only by integrating microscopic observations with biological validation will it be possible to determine whether NL represents merely an ultrastructural phenomenon or a clinically meaningful determinant of endodontic treatment outcomes. Until such evidence becomes available, NL should be regarded as a promising experimental research concept rather than a validated clinical endpoint or predictor of endodontic success.

Our results showed that current evaluations of NL lack standardized methodologies, including accepted aging protocols and consistent sample handling procedures, particularly regarding tracer application. Furthermore, testing conditions across different materials are highly heterogeneous, compromising the standardization and comparability of NL measurements. Importantly, the absence of internationally accepted standards for NL assessment currently precludes objective ranking of individual experimental methodologies. Different aging strategies reproduce distinct degradation mechanisms, whereas different tracer systems evaluate different physicochemical or biological processes. Consequently, methodological diversity should not be interpreted simply as variation in study quality, but rather as evidence that the field has not yet reached consensus regarding the most biologically relevant experimental model. Until standardized protocols become available, direct comparisons among studies should be interpreted with caution. It is crucial to recognize that leakage is not an abrupt phenomenon but rather the result of gradual changes, typically associated with material degradation or interfacial compromise. Therefore, disregarding NL in recently placed materials may reduce clinical benefit. In this context, the implementation of rigorous aging protocols designed to simulate extreme conditions should be considered not merely optional but essential.

It has been stated that, to be clinically relevant, *in vitro* adhesive studies should report no less than 3 months of aging and make use of simulated functional forces (masticatory simulator) (15). Importantly, loss of bond strength is first detectable *in vitro* at 3 months (70), and interfacial leakage increases as the bond degrades (71). Notably, it has been reported that functional forces contribute to the degradation of the resin bond (70, 72). However, among the 45 studies included in this article, the aging process was one of the most heterogeneous methodological aspects observed. Aging protocols ranged from studies reporting only 24 h of aging to others employing several months of storage, including more controlled procedures using thermocycling (TC). Although TC is a method conventionally used to simulate the thermal changes and water exposure that may occur in the oral cavity (during eating, drinking, or even breathing) (73), concerns over its ability to simulate intra-oral aging remain (74).

Notably, TC represents an important artificial aging procedure because it combines hydrolytic and thermal degradation, which is believed to produce debonding (75, 76); additionally, some researchers have used thermomechanical aging, which incorporates mechanical loading cycles (77). Importantly, leakage increases with thermal stress, and it has been estimated that 10,000 cycles represent one-year oral function, although no consensus has been reached (73). Moreover, it has been suggested that the number of cycles is often selected for convenience (77), though ISO (The International Organization for Standardization) recommends 500 cycles between 5 °C and 55 °C (78). Unfortunately, although the importance of controlled aging process is clear; some studies did not report this aspect at all. The wide variety of variables and approaches related to aging requires that all collected data be interpreted with caution, particularly considering the impact that each independent aging strategy may have on the obtained results and their subsequent clinical extrapolation. For future NL studies, establishing a standardized aging method that considers the particularities of the nanometric environment is mandatory.

None of the included studies reported the use of a masticatory simulator. This is a biomimetic mechanical device that replicates human chewing movements, biting, tongue movement, and contraction of the masticatory muscles, and can even generate human-like food boluses by usingbiomimetic masticatory components and advanced sensors and control systems. Additionally, it typically incorporates artificial saliva (79). In this regard, the use of this device would afford a sound simulation of oral physiology, which may have an inherent deleterious effect on NL. However, there are currently no standardized methods and criteria evaluating its stability and reliability (79, 80), which could impact their use in *in vitro* research.

Another crucial aspect is the high number of publications relying on qualitative models designed to microscopically identify NL through particle detection. These models often assume or dismiss the presence of NL based solely on the visualization of such nanostructures at potential interfaces between materials and dental tissues. Most *in vitro* studies in endodontics rely on experimental models based on extracted teeth, in which root canals are instrumented and subsequently obturated to evaluate the sealing ability of different materials. In qualitative *in vitro* research approaches, tracer particles (such as silver nanoparticles) are frequently used and later identified through electron microscopy techniques. However, this type of qualitative methodology may introduce an important source of bias: the overinterpretation of isolated observations obtained from selected microscopic images. Such interpretations may lead to definitive assumptions regarding the presence or absence of NL, which, from an observer-dependent standpoint, may be considered overstated. To better illustrate the scale of the phenomenon under investigation, a conceptual analogy can be proposed: consider a root canal approximately 16 mm in length, prepared using endodontic instrumentation to achieve relatively standardized diameters, resulting in a geometry that can be approximated as a truncated cone (Figure 3a). Nanometric analysis techniques may be applied to determine whether a nanoparticle is capable of penetrating this structure; if this same system were proportionally scaled to macroscopic dimensions (for instance, comparable to the size of the Eiffel Tower in Paris, France), detecting a single nanoparticle would be analogous to drawing conclusions based on the observation of a human hair located at the base of such a structure (Figure 3b). From this perspective, the substantial disparity in scale becomes evident, highlighting the inherent complexity of interpreting results based solely on qualitative observations at the nanometric level.

**Figure 3:**
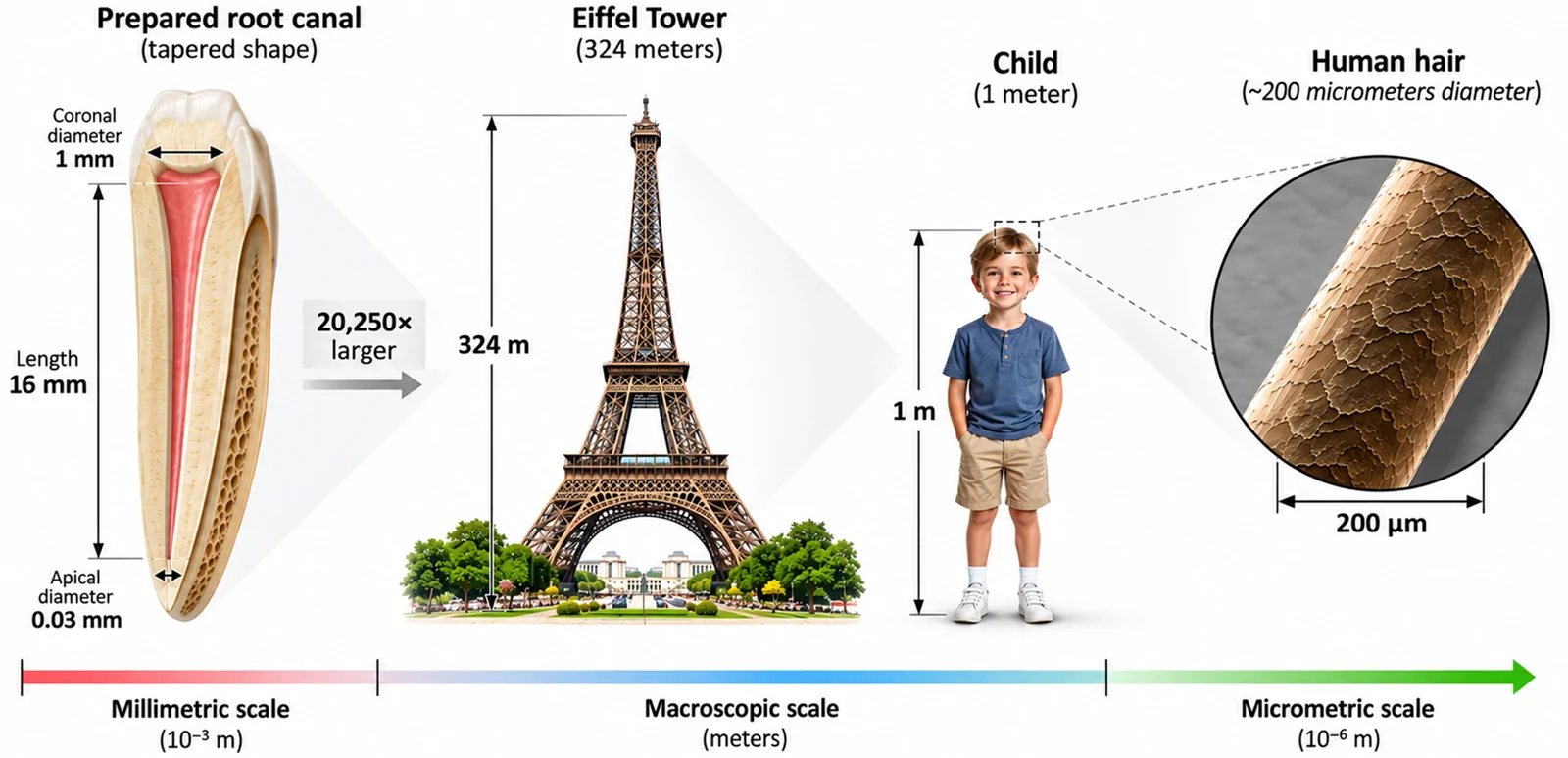
Conceptual analogy illustration of the nanoleakage phenomenon. **(a)** The approximate dimensions of a prepared root canal. **(b)** Detecting a single nanoparticle would be analogous to drawing conclusions based on the observation of a human hair located at the base of the Eiffel Tower.

To address the limitations associated with qualitative research, dynamic fluid-based models have been proposed. In this context, NL can be quantified in terms of flow rate (nanoL per second, nL/s) using the so-called nanoflow device (26, 27). This system detects bubble movement through an optical sensor with a resolution of 0.196 nL or greater in real time (26, 27, 40). However, although fluid-based approaches represent a promising alternative, the behavior of the liquid medium and its chemical interactions may also introduce bias. In the assessment of NL, both the size of the tracer and the physicochemical properties of the mobile phase are critical, as they influence the ability to detect or infer the presence of nanoporosities. If the solution is chemically incompatible with the environment, or if its rheological properties affect passive penetration, an additional source of bias may be introduced. A promising option can be to analyze the flowability of not only liquids but of inert gases like nitrogen, while an alternative was proposed as pilot studies to analyze the gas permeability of endodontic biomaterials. Although this alternative can be particularly interesting, its methodological complexity is still an aspect that requires analysis (81, 82).

It must be emphasized that in Sano et al.'s seminal articles, SEM and TEM with silver nitrate (3 mol/L solution for 24 h) as a tracer were used, and NL was expressed in % by measuring said tracer within the hybrid layer, whereby “Global leakage scores of each specimen were calculated as the percent of the total cut dentin surface that was penetrated by silver nitrate.” (20, 21). Notably, of the identified studies that evaluated NL at the interface between restorative materials and adhesive systems in coronal dentin, only 5 used SPP (33, 34, 47, 53, 54) to simulate a more realistic scenario when bonding to a vital tooth. SPP provides water and interferes with resin polymerization. Indeed, studies have demonstrated the adverse effect of pulpal pressure on the dentin bond strength (83, 84), and future studies evaluating NL at this interface should include the SPP model. Interestingly, only two of the included studies refer to the evaluation of NL in orifice barriers (23, 35). These barriers are placed as sub-seals following root canal treatment to resist microbial leakage if the primary coronal seal fails, with a recent systematic review of *in vivo* studies confirming this role (85).

On the other hand, nano-computed tomography (nano-CT) has been used to analyze the ultrastructure of cartilage and bone, materials behavior, and vascular networks (86–88). Nano-CT uses a nano-focal spot source (< 400 nm), thus providing ultra-high spatial resolution (89), and has been reported as a promising technology in the field of restorative dentistry (90) and endodontics, providing faster scanning, higher precision, and minor voxel size when compared with micro-CT (91, 92). Notably, while nano-CT has been used to characterize dental composites (93), some researchers have already employed nano-CT along with radiopaque tracers to evaluate NL expression at the dentin/resin composite interface, as well as to analyze polymerization shrinkage (94). However, nano-CT has not been extensively used to study NL expression, perhaps owing to high expenses and restricted access to this technology, or to its small volume of interest—inherently smaller than micro-CT—which may affect the reliability of obtained results (89). There is a dire need to further explore this technology to study NL expression in order to better recognize its precision when evaluating NL.

It should be noted that three of the included studies must be interpreted with caution, since the authors used conventional light microscopy to characterize NL (Table 1). Although this represents a methodological limitation when compared with more sensitive techniques such as SEM or TEM, at the time these articles were published, the authors may have assumed that the use of nanometric tracer molecules was sufficient to identify the presence of NL. In this regard, it is important to consider the limited spatial resolution of light microscopy, particularly because axial resolution is several times poorer than lateral resolution (95). Indeed, it should be remembered that “because the wavelength of blue light is 400 nm, the theoretical resolution of a conventional optical microscope can never be higher than 200 nm” (96). Due to this important limitation, such findings should be considered with caution, as conclusive evidence could not be reliably reproduced under current methodological standards.

Several limitations should be acknowledged in the present narrative review. Inherently, formal risk-of bias assessment or quantitative evidence grading was not performed. Also, even when multiple databases were consulted, additional studies referring to NL may exist in other scientific databases. Despite this restriction, the present review provides a critical framework for interpreting the available evidence, including the potential overestimation of the clinical and methodological significance of NL findings. An additional limitation of this analysis relates to the relatively recent introduction and conceptual consolidation of the term NL. It is possible that previous investigations from different research fields may have indirectly evaluated NL phenomena without explicitly recognizing or defining them as such, potentially categorizing these observations under the broader concept of conventional ML. This issue underscores the need for greater conceptual standardization and may justify future investigations exploring historical leakage models as well as the application and interpretation of NL across other dental disciplines. Finally, to establish a more standardized and clinically meaningful approach for assessing NL, future studies should provide more detailed analyses of aging protocols, simulated clinical conditions, tracer types and concentrations, immersion times, microscopic analysis techniques, and, most importantly, quantitative and reproducible assessment methods.

## 5 Conclusions

Understanding NL is an important goal in endodontics, yet models are underdeveloped. Existing evidence is limited by methodological inconsistencies and a lack of clinical validation. Future research should prioritize the development of dynamic, clinically relevant models to better understand interfacial sealing and improve the predictability of endodontic outcomes, especially considering that NL occurs through submicron spaces, even in the presence of gap-free restorations and sound clinical practice.
